# Cloacal Exstrophy with Mature Teratoma: A Rare Association in a Neonate

**Published:** 2016-04-10

**Authors:** Prashant Sadashiv Patil, Paras Kothari, Abhaya Gupta, Rahul Gupta, Geeta kekre, Vishesh Dikshit, Ravi Kamble

**Affiliations:** Department of Paediatric Surgery, Lokmanya Tilak Municipal Medical College and General hospital, Mumbai, Maharashtra.

**Keywords:** Cloaca, Exstrophy, Mature Teratoma, Neonate

## Abstract

Cloacal exstrophy is a very rare and complex malformation. We report a neonate of cloacal exstrophy with mature teratoma presenting as a component of exstrophy. To our knowledge this has not been reported in the literature.

## CASE REPORT

A female full term baby (2.6 kg) was born by caesarean section to a 25-year-old primigravida with no antenatal history of any infection, drug intake or radiation exposure to mother. The marriage was non consanguineous. The baby cried immediately after birth. APGAR score was 8/10. Examination showed stable vital parameters, presence of a protruding and exposed bowel and bladder plates in the lower part of abdomen and perineum. Urine was draining from left ureteric orifice in the bladder plate and meconium seen coming from the bowel plate. There was a hair bearing structure on right side of bowel plate and another cystic structure in the perineum continuous with it. There were no palpable gonads or phallus like structure seen (Fig. 1). Systemic examination of baby was normal. Spine x-ray revealed normal spine with radio-opaque mass in the pelvis (Fig. 2). Ultrasound of abdomen showed prominence of right pelvicalyceal system and dilated left ureter. A mass was seen in presacral region in midline in pelvis. USG spine was normal for posterior vertebral elements, para-spinal muscles, and spinal cord. Echocardiogram was normal. 

**Figure:**
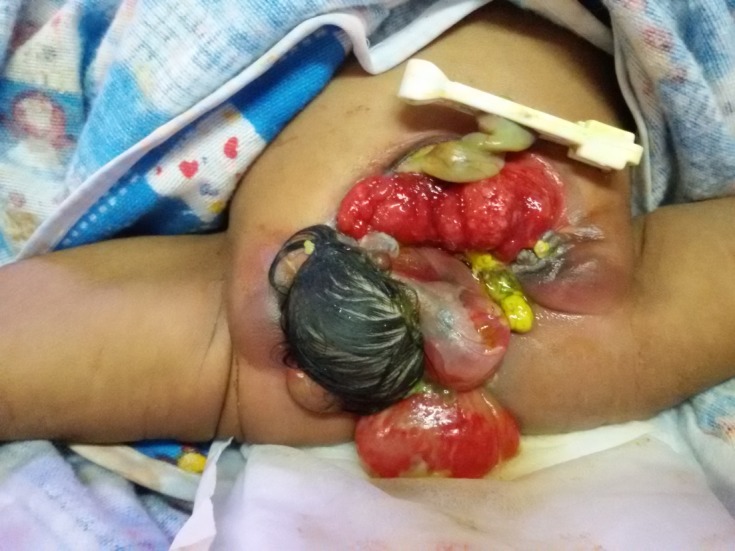
Figure 1: Cloacal exstrophy and teratoma

**Figure:**
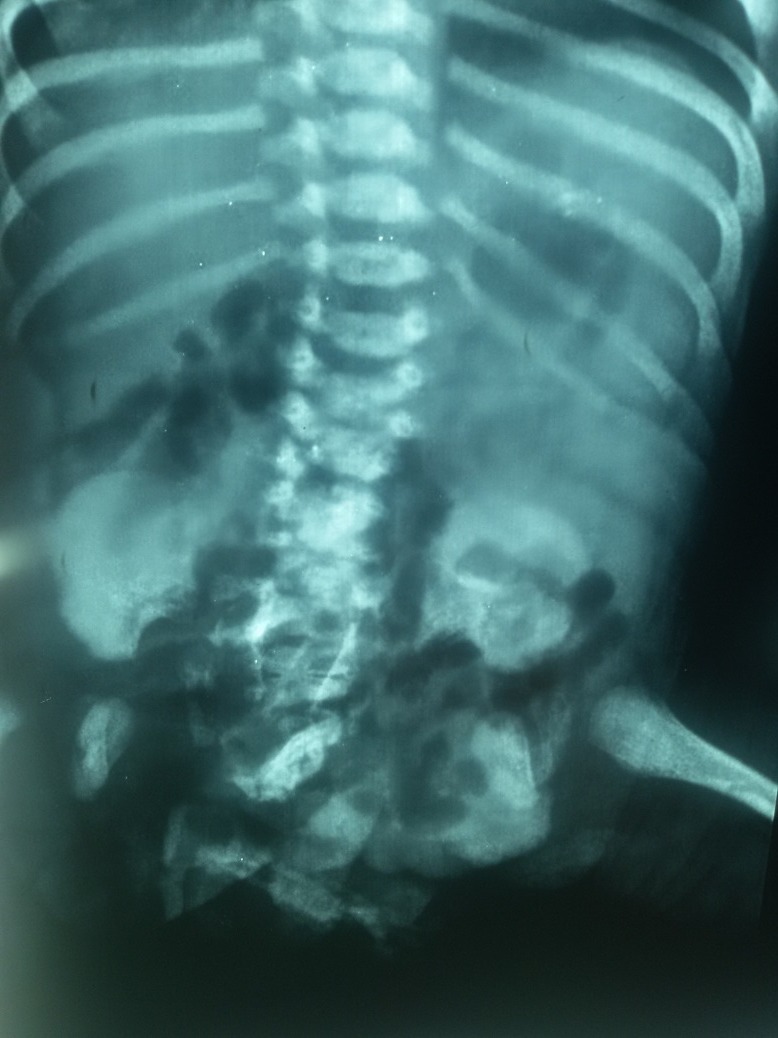
Figure 2: X-ray showing bony opacities in the pelvis

Immediate management was directed to stabilization of the infant and the bowel and exposed tissues were covered with warm saline soaked gauze and protective plastic dressing. After counseling of parents regarding guarded prognosis and staged repair, baby was taken for surgery on day of life 3. 

Operative steps: Bilateral ureteric orifices were cannulated with 5fr infant feeding tube. Teratoma component had parts of head like structure with hairs, cyst like structures and a bony component which was attached to anterior part of sacrum. Soft tissue component and easily accessible part of bony component were removed. Bony component could not be removed completely due to close proximity to sacrum. Bowel plate freed from the bladder plate and fashioned into end colostomy leaving the patient with whole of small intestine, ileocecal junction, cecum, appendix and part of ascending colon. The two halves of bladder plate separated and then joined in the midline creating the posterior wall of bladder. Right and left hemivaginal openings were kept in the bladder plate below openings of bilateral ureters. Histopathological examination of presacral mass revealed glandular structure, mesenchyme with vessels, smooth muscles, skeletal muscles, adipose tissue. No immature element seen. Separately sent bony tissue showed bony trabeculae with hematopoietic marrow. These features were suggestive of mature teratoma.

Patient is on regular follow up for 18 months. Patient has been admitted several times for failure to thrive. At age of 2 years baby weighs 6.5 kg, can stand and walk without support. MRI pelvic floor muscles showed atrophic coccygeus muscle while other pelvic diaphragm muscles were not appreciated. Considering poor general condition and minimal chance of urinary and fecal continence after any definitive procedure patient is being managed conservatively.

## DISCUSSION

During the fourth and seventh weeks of gestation, the cloaca is subdivided by the urorectal septum to form the anorectal canal and the primitive urogenital sinus.[1] A simultaneously occurring sacrococcygeal teratoma could encroach between the layers of the cloacal membrane and prevent descent and fusion of the urorectal septum to the cloacal membrane. An anterior location of the tumor could thus result in the absence of rectum and anus. The physical presence of a teratoma could also prevent fusion of the genital folds, resulting in a bifid scrotum and hypospadias [2] in male neonate. In our case this same mechanism could be responsible for the external appearance of bifid bladder plates, anorectal malformation, bifid vagina with part of tumor protruding externally. The massive growth of teratoma in presacral space could have hindered the development of Mullerian structures in our patient. 

Cloacal exstrophy is associated with many other anomalies including cardiovascular, central nervous system, omphalocele (70-90%), vertebral anomalies (46%), upper urinary tract (42%), malrotation (30%), lower extremity anomalies (30%), double appendix (30%), absent appendix (21%), short small bowel (19%), small bowel atresia (5%), and abdominal musculature deficiency (1%) [3]. Upper urinary tract anomalies include pelvic kidney, horseshoe kidney, hypoplastic kidney and solitary kidney [4]. Vertebral malformations include sacralization of L5, congenital scoliosis, sacral agenesis, and interpedicular widening. To the best of our knowledge association of cloacal exstrophy with sacrococcygeal teratoma has not been described in literature.

Immediate management of exstrophy patients is directed to the medical stabilization of the neonate. For infants who have minor associated malformations and are medically stable, staged closure can be considered within 48-72 hrs after birth. Evaluation of the genitalia and gender assignment should be made by a gender assignment team, including a pediatric urologist, pediatric surgeon, pediatrician, and pediatric endocrinologist. The initial operation consists of separating the bowel from the bladder to create an intestinal stoma; closing the omphalocele; and re-approximating, closing, or leaving the exstrophied bladder undisturbed. In our case, management of teratoma was additional challenge as it was large and extending deep in to the pelvis. We excised the teratomatous component as much as possible thereby helping the bladder plates to get approximated. Bilateral hemivaginal openings kept in bladder plate below ureteric openings. Hindgut segment was brought out as end colostomy thus avoiding ileostomy and its complications.

## Footnotes

**Source of Support:** Nil

**Conflict of Interest:** Nil
